# Case report: Clinical and genetic features of pediatric choroidal melanoma

**DOI:** 10.3389/fmed.2024.1480111

**Published:** 2025-03-13

**Authors:** Paola Valente, Angela Galardi, Angela Di Giannatale, Antonino Romanzo, Antonio Novelli, Valeria Orlando, Marta Colletti, Ida Russo, Rita De Vito, Giancarlo Iarossi, Sergio Petroni, Lorenzo Sinibaldi, Luca Buzzonetti

**Affiliations:** ^1^Ophtalmology Unit, IRCCS, Ospedale Pediatrico Bambino Gesù, Rome, Italy; ^2^Hematology/Oncology and Cell and Gene Therapy Unit, IRCCS, Ospedale Pediatrico Bambino Gesù, Rome, Italy; ^3^Translational Cytogenomics Research Unit, Bambino Gesù Children’s Hospital, IRCCS, Rome, Italy; ^4^Laboratories, Pathology Unit, IRCCS, Ospedale Pediatrico Bambino Gesù, Rome, Italy; ^5^Medical Genetics Unit, Bambino Gesù IRCCS Pediatric Hospital, Rome, Italy

**Keywords:** uveal melanoma, GNAQ gene, oncology, pediatric, cancer

## Abstract

Uveal melanoma (UM) is the second most common type of primary melanoma in adults, but it is extremely rare in children. We report a 12-year-old boy with a rare juvenile case of UM characterized by specific clinical and genetic features, including eye imaging and cytogenetic analysis. The tumor was analyzed using immunohistochemistry in order to confirm the clinical diagnosis and using next-generation sequencing (NGS) in order to investigate the correlation between pathological features and prognosis. The NGS revealed a somatic mutation in the GNAQ gene. Furthermore, we established a primary cell line (Opbg-UM1) to better understand the biology of this tumor in the pediatric setting. However, our case identified several factors predictive of poor prognosis, such as tumor proximity to the fovea and optic disc, large size, lack of pigmentation with mushroom configuration in category T2, and a complex karyotype showing numerical abnormalities on chromosome 6 and a mosaic loss of the Y chromosome in blood and in the primary cell line. This mutation may represent a poor prognostic factor in older children with UM.

## Introduction

Uveal melanoma (UM) is the most common intraocular malignancy in adults, affecting the elderly population with an average age of 58 years. However, UM with an averege age at diagnosis, although it is very rare in childhood (1, 2). A comprehensive analysis of 8,101 patients with UM, identified at the Wills Eye Hospital Oncology Service over a 38-year period, revealed that 122 (1.5%) were young patients aged 20 years or less (3). A study based on the Finnish population found that the frequency of UM in patients <25 years of incidence age was 1.3%, whereas in those ≤20 years of age, it was only 0.6% (4). Children affected by UM usually have a better prognosis compared to adults; this difference may depend on the tumor size and other different clinical features such as the histopathological and tumor’s molecular characteristics, along with the overall response to treatment. Additionally, age-related variations in the immune system and the genetic makeup of pediatric patients may contribute to the different outcomes (1). In adults, chromosomal alterations such as monosomy of chromosomes 3 and 8q are associated with a poor prognosis (5). Several risk factors predisposing to UM include fair skin, light-colored eyes, congenital ocular melanocytosis, neurofibromatosis type 1, and the BAP1-tumor predisposition syndrome (6).

## Case description

**Figure 1:**
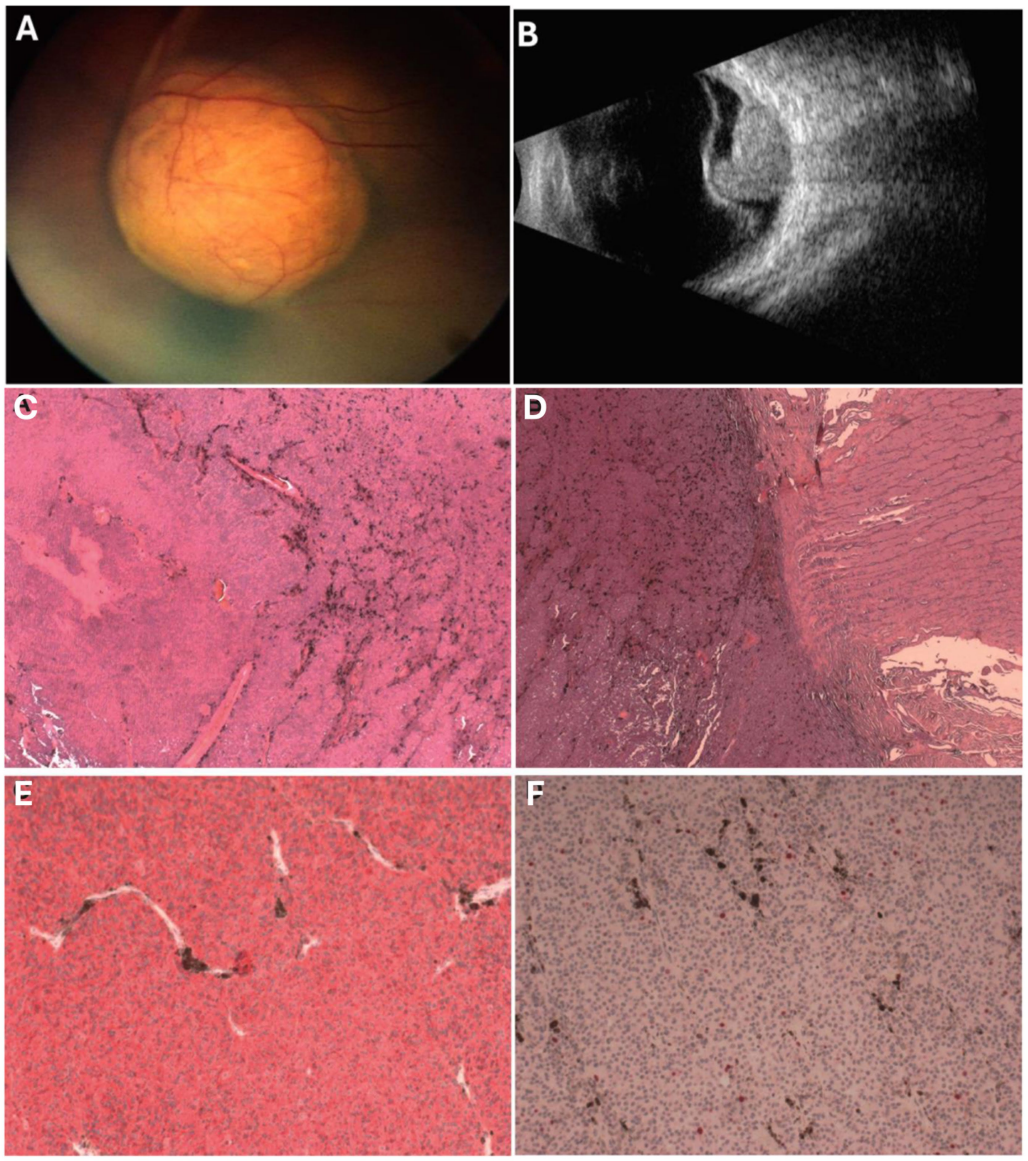
**(A)** Amelanotic mass overhangs the optic disc and macula associated with extensive exudative retinal detachment; **(B)** Mushroom-shaped solid mass, overhangs optic nerve and macula, dishomogeneous internal structure, medium internal reflectivity. **(C)** Hematoxylin and Eosin (H&E) shows tumor mainly composed of spindle type A and B melanoma cells with no significant mitotic activity, no necrosis (H&E, 5x). **(D)** Pre-laminar optic nerve invasion, surgical cut end tumor free (H&E, 5x). **(E)** The cells stained positive for the melanocytic marker MART-1 (MART-1, 10x). **(F)** The proliferation index was <5% in melanoma cells with no clusters of proliferating cells (Ki67, 10x).

A 12-year-old boy was referred to our hospital for blurred vision in the left eye. His medical history was unremarkable. The patient had no family history of melanoma or other genetic diseases. On ophthalmological examination, visual acuity was 20/20 in the right eye and light perception was normal the left. Fundus ophthalmoscopy showed a melanotic mass in the left eye overlying the optic disc and macula, associated with extensive retinal detachment (Figure 1). Ultrasonography revealed a mushroom-shaped solid mass, measuring 11.54 mm in basal diameter and 8.05 mm in thickness, overhanging the optic nerve and the macula, with an inhomogeneous internal structure and medium internal reflectivity (Figure 1). Fluorescein angiography showed irregular hyperfluorescence in the early phase and late phase staining, with the vascular network clearly represented. We performed magnetic resonance imaging (MRI), which showed a nodular mass at the posterior pole of the left eye that was hypointense on the T2-weighted images and had strong contrast enhancement on post-contrast T1-weighted images. A diagnosis of UM was strongly suspected, and the patient underwent enucleation with the placement of an endoprosthetic implant. From the tumor in the enucleated eye, we established a cell line that we named the Opbg-UM1 cell line. Written and verbal informed consent was obtained from the patient’s parents.

Gross examination without retinal and scleral extensions of the enucleated eye revealed a mushroom-shaped pigmented lesion and retinal detachment. Histologically, the tumor consisted mostly of spindle cells growing in a compact, cohesive fashion with rare intercalated epithelioid cells. The neoplastic cells showed low mitotic activity (1/40 high-power field) without evidence of atypical mitosis or nuclear inclusions. The vascular pattern showed parallel vessels without lakes and loops. There was no evidence of the presence of a pre-existing nevus (Figure 1).

Molecular characterization of blood DNA and tumor tissue was performed using next-generation sequencing (NGS) using the Twist Custom Panel kit (clinical exome − Twist Bioscience) on the NovaSeq6000 platform (Illumina). Specifically, BAP1, CYSLTR2, GNA11, GNAQ, SF3B1, BRCA2, CDK4, CDKN2A, MITF, POT1, PTEN, RB1, and TP53 were sequenced. The somatic c.626A > T variant in the GNAQ gene in mosaic form (~ 55%) was found in the tumor; no pathogenic germline variants were detected.

**Figure 2:**
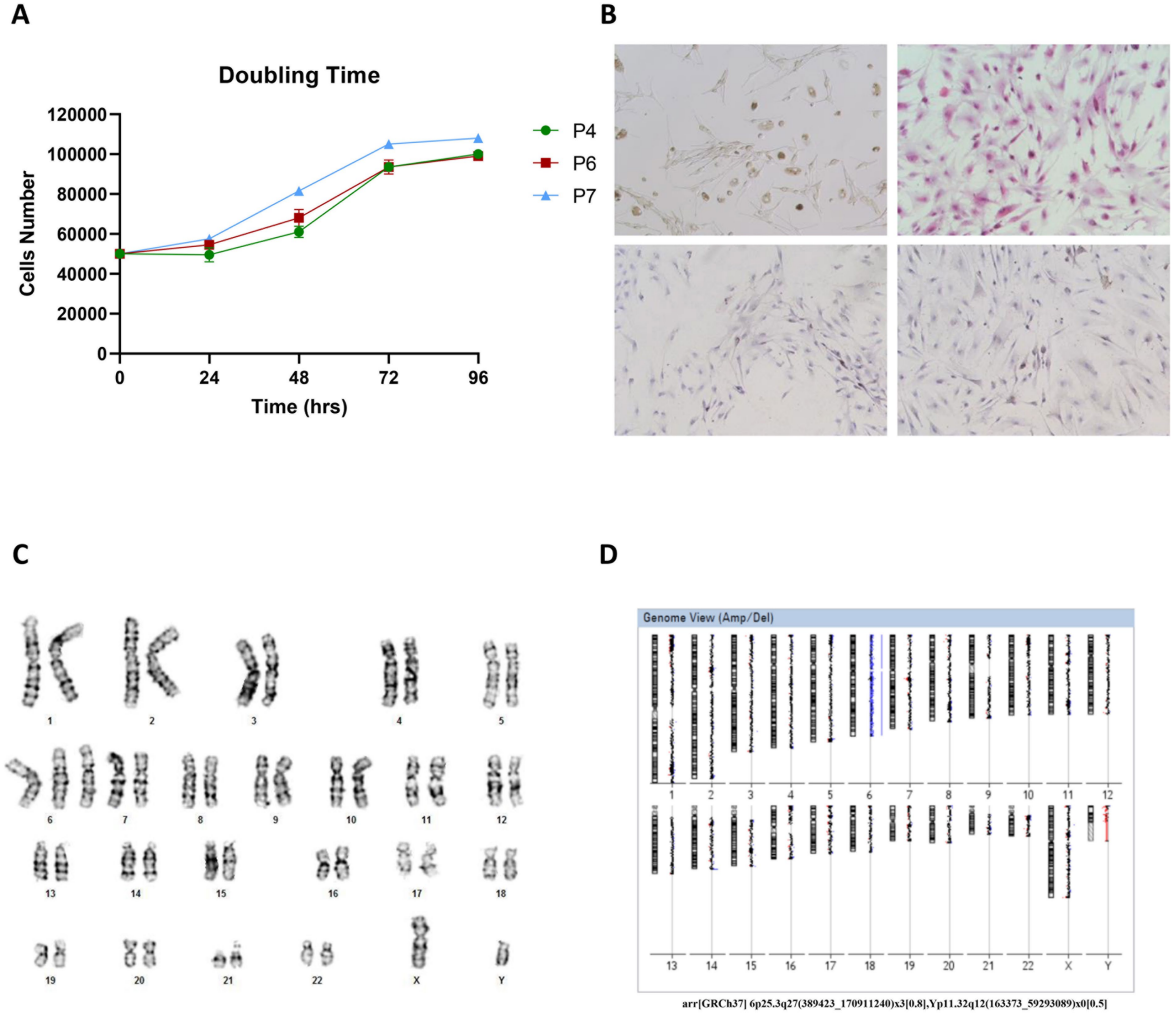
**(A)** The population doubling time was calculated as reported and found to be of about 72 h regardless of the initial passages (4, 6 or 7), cells were plated and counted at 24 h, 48 h, 72 h, and 96 h. **(B)** IHC characterization of Opbg-UM1 cell line. Light microscopy revealed Opbg-UM1 cell in Bright Field (top left): the cultured cells showed high nuclear/cytoplasmic ratios, large prominent nucleoli, and diffuse deposition of chromatin throughout the nucleus and positivity for H&E (top right), HMB-45 (bottom left) and MART-1 (bottom right) (magnification 20×). **(C)** Cytogenetics analysis of the tumor cells revealed a complex karyotype. The modal chromosome number of the cultured cells was 47. Numerical abnormalities of chromosome 6. The complete karyotype of cell: 47, XY, +6. **(D)** Report from array-CGH analysis software, showing the chromosome 6 trisomy and loss of chromosome Y both in mosaic. arr[GRCh37] 6p25.3q27(389423_170911240)x3[0.8], Yp11.32q12(163373_59293089)x0[0.5].

We then characterized the Opbg-UM1 cell line by its proliferative capacity at different passages (4, 6, and 7). The growth curve displayed a doubling time of approximately 72 h regardless of the initial passages (Figure 2). Karyotype analysis of metaphases obtained from melanoma cell cultures revealed a mosaic trisomy of chromosome 6 (80%) and a mosaic loss of chromosome Y (50%). Array-based CGH confirmed these results and excluded the presence of copy number variants (CNVs) in the tumor cells (Figure 2).

The immunohistochemistry of UM cells showed positivity for MART-1, HMB-45, and Ki-67 (Figure 2). The cells were characterized by a high nuclear/cytoplasmic ratio, large prominent nucleoli, and diffuse deposition of chromatin.

The patient is now in good health, without disease after 5 years of follow-up.

## Discussion

UM is a rare finding in children. The differential diagnosis of amelanotic melanoma in young patients includes chorioretinal granuloma, choroidal osteoma, lymphoma, and astrocytic hamartoma. Chorioretinal granuloma typically presents as a yellow-white mass and is often associated with systemic inflammatory diseases such as sarcoidosis. Osteoma is a yellow-orange ossifying lesion that is generally benign in nature. Choroidal lymphoma may appear as an amelanotic choroidal infiltration that obscures the underlying vessels. Astrocytic hamartoma generally manifests as creamy-white, multilobulated lesions, typically associated with tuberous sclerosis and occasionally with neurofibromatosis. In 1991, Shields et al. reported a series of 40 children with UM, with a cumulative survival rate of 96% at 5 years (7). In 2012, Shields et al. reviewed 8,033 cases of UM, of which 108 were children. They identified that compared to adults, pediatric UM, is more likely to have an iris location, smaller tumor size, greater frequency of tumor pigmentation, greater distance from the macula and optic disc, and less frequent extraocular extension and metastasis (1). Regarding prognosis, Kaliki et al. matched three groups based on gender, tumor location, location of anterior margin, tumor thickness, tumor basal diameter, and extrascleral extension. Their findings indicated that the metastases estimated at 3, 5, and 10 years were 1, 8, and 8% in children compared to 13, 16, and 24% in adults (8). In 2016, “The Pediatric Choroidal and Ciliary Body Melanoma Study,” a retrospective multicenter observational study based on 114 children and 185 young adults, reviewed the prognosis factors. Children younger than 18 years had a more favorable prognosis compared to young adults (18–24 years old), and the TNM stage is a predictor of survival. Moreover, men had a better survival prognosis when compared with women (100% vs. 85%). Congenital melanocytosis is associated with increased mortality, with a 5.6 times higher risk of metastasis in young patients (9).

Shields et al. evaluated the clinical features and prognosis of posterior UM based on the American Joint Committee on Cancer (AJCC) classification. They found that factors for metastasis in category T2 were increasing age (*p* < 0.001), mushroom configuration of tumor (*p* = 0.004), and amelanotic tumor (*p* = 0.003) (10). Recently, genome analysis has gained increasing importance in prognosis. Chromosomal alterations such as monosomy of 3, loss of 1p, 6q, 8p, 9p, and gain of 1q, 6p, and 8q have been recognized as prognostic factors in UM (11). Monosomy of chromosome 3 occurs in almost half of UM and is the most significant prognostic chromosomal marker.

Numerical abnormalities on chromosome 6, either the gain of 6p or the loss of 6q, are commonly found in UM. These genetic changes are linked to the upregulation of certain genes that may contribute to the uncontrolled growth of melanoma cells and are typically linked to disease progression and prognosis (12). Furthermore, losses on chromosome 6q may involve the deletion of tumor suppressor genes, further promoting the development and progression of UM (13).

Trisomy 6, together with other abnormalities of chromosome 6, has been described in neoplastic tissues, such as Merkel cell carcinoma (14).

Our case showed clinical, histopathological, and karyotype characteristics predictive of a more favorable prognosis, such as age (younger than 18 years), gender (male), no ocular melanocytosis, spindle cell type histology, absence of necrosis, and absence of chromosome 3 monosomy. On the other hand, some clinical and genetic features, such as tumor proximity to the fovea and optic disc, large size, and lack of pigmentation, are unusual for a UM in children. Furthermore, a poor prognosis was suggested because it was an amelanotic tumor with a mushroom configuration in the T2 category (11).

In the present case, a karyotype with numerical abnormalities on chromosome 6 was revealed. Moreover, NGS revealed a somatic mutation in the GNAQ gene. Van Raamsdonk et al. described the frequent somatic mutation of the GNAQ gene and its role in UM oncogenesis (14, 15). The GNAQ gene encodes members of the q class of heterotrimeric G-protein *α* subunits. GNAQ mutations can result in constitutive G-protein activation, leading to a signaling event cascade. Active GNAQ upregulates the MAP kinase pathway. Mutations in GNAQ can result in the activation of these signaling pathways, promoting uncontrolled cell growth and survival as key features of cancer cells. Van Raamsdonk et al. described these mutations in codons 209 and 183 of GNAQ (16). The mutation in codon 209 was observed in 44.8% of primary UM and 21.7% of UM metastases, while the mutation in codon 183 was observed in 2.8% of primary UM and 5.9% of UM metastases. In our study, the tumor tissue showed the missense variant c.626A > T, which at the protein level determines the amino acid change p.Gln209Leu (rs121913492). This specific mutation refers to a change in the DNA sequence at position 626, where adenine (A) is replaced by thymine (T). The variant is reported in the ClinVar database (ID: 375955) and can be classified according to the American College of Medical Genetics and Genomic (ACMG) guidelines as a possibly pathogenic variant (class 4). Several studies suggest that these oncogenic mutations in GNAQ are detected early in the progression of UM (16). Karyotype analysis of tumor cells also revealed another important finding: a mosaic loss of chromosome Y. Involvement of chromosome Y has been rarely detected in UM, and an association between a mosaic loss of the Y chromosome and cancer susceptibility has been reported (17). The presence of a mutation in the GNAQ gene, a karyotype with a mosaic trisomy of chromosome 6, and a mosaic loss of chromosome Y may play a role in the malignancy grade of the tumor in older children. The combination of these genetic alterations suggests a heterogeneous genetic profile, potentially contributing to the development or progression of cancer. Understanding these genetic variations is crucial for personalized medicine and tailored treatments in UM.

## Data Availability

The original contributions presented in the study are publicly available. This data can be found here: https://www.ebi.ac.uk/eva/?eva-study=PRJEB85793 accession number del dataset: PRJEB85793.
